# GLP-1 and Intestinal Diseases

**DOI:** 10.3390/biomedicines9040383

**Published:** 2021-04-05

**Authors:** Jenna Elizabeth Hunt, Jens Juul Holst, Palle Bekker Jeppesen, Hannelouise Kissow

**Affiliations:** 1 Department of Biomedical Sciences, Faculty of Health and Medical Sciences, University of Copenhagen, 2200 Copenhagen, Denmark; jenna.hunt@sund.ku.dk (J.E.H.); jjholst@sund.ku.dk (J.J.H.); 2 Novo Nordisk Foundation Center for Basic Metabolic Research, Faculty of Health and Medical Sciences, University of Copenhagen, 2200 Copenhagen, Denmark; 3 Department of Medical Gastroenterology and Hepatology, Rigshospitalet, 2200 Copenhagen, Denmark; palle.bekker.jeppesen@regionh.dk

**Keywords:** GLP-1, intestinal disease, short-bowel syndrome (SBS), mucositis, inflammatory bowel disease (IBD), coeliac disease

## Abstract

Accumulating evidence implicates glucagon-like peptide-1 (GLP-1) to have, beyond glucose maintenance, a beneficial role in the gastrointestinal tract. Here, we review emerging data investigating GLP-1 as a novel treatment for intestinal diseases, including inflammatory bowel diseases, short-bowel syndrome, intestinal toxicities and coeliac disease. Possible beneficial mechanisms for these diseases include GLP-1′s influence on gastric emptying, its anti-inflammatory properties and its intestinotrophic effect. The current knowledge basis derives from the available GLP-1 agonist treatments in experimental animals and small clinical trials. However, new novel strategies including dual GLP-1/GLP-2 agonists are also in development for the treatment of intestinal diseases.

## 1. Introduction

Intestinal disease describes the pathological changes that may occur in the intestine from the duodenum to the rectum. The term encompasses a broad spectrum of acute and chronic conditions, including inflammatory bowel disease (IBD), adverse effects of cytotoxic treatments and coeliac disease, which will be explored in this review. Central to intestinal disease recovery is the re-establishment of the intestinal epithelial area and integrity [1,2]. Incomplete recovery can lead to a decrease in absorptive capacity and barrier function [2]. Additionally, severe cases may require surgical resection, which can lead to malabsorptive conditions such as short-bowel syndrome (SBS) and/or intestinal failure, which will also be explored in this review. In addition to patient suffering, intestinal diseases have a high economic impact on healthcare systems due to hospitalizations, patient care and pharmaceuticals, alongside the loss of productivity and decreased quality of life [3,4,5].

Current treatment strategies of intestinal diseases that impede absorption focus on symptom relief, but ultimately, treatments strive to restore the mucosal function and finally to improve quality of life [6,7]. Recently, emerging evidence has suggested that glucagon-like peptide-1 (GLP-1) could be a candidate for treating intestinal-related diseases such as IBD, intestinal mucositis, coeliac disease and SBS. At present, GLP-1 mimetics are approved for the treatment of diabetes and multiple drugs are in clinical use [8]. These drugs have undergone rigorous safety testing, with minimal serious adverse effects, and could provide an attractive alternative for the treatment of intestinal diseases.

In this review, a detailed literature search on PubMed was conducted to collate the latest developments in the field of intestinal diseases and GLP-1. First will be a summary of GLP-1 and its physiological effects. Next will be a discussion of the latest developments in GLP-1 treatment of IBD, SBS, intestinal toxicity and coeliac disease. Third will be an evaluation of GLP-1’s theoretical safety. Finally, we present a discussion of the future directions in the field of GLP-1 and intestinal diseases.

## 2. Biosynthesis of GLP-1

GLP-1 is a post-translational product of proglucagon [9]. Proglucagon is encoded by the GCG gene, which is expressed in the alpha cells of the pancreatic islets, the enteroendocrine cells of the gut epithelium and the neurons of the brain stem, and it is processed in a tissue-specific manner by prohormone convertases to generate differential post-translational products. In the pancreatic α-cell, the actions of prohormone convertase 2 results in the formation of glucagon, glicentin-related pancreatic polypeptide (GRPP) and the so-called “major proglucagon fragment” [10]. In a small group of neurons in the nucleus of the solitary tract (NTS) of the brain stem and the so-called L-cells, the endocrine cells of the intestinal epithelium, the presence of prohormone convertase 1/3 results in the formation of glicentin (which may be further processed to form oxyntomodulin), GLP-1, intervening peptide-2 (IP2) and GLP-2 [10]. From the gut, the proglucagon-derived products, GLP-1 and GLP-2, are released in parallel in a nutrient-dependent manner [11,12,13]. The L-cells are distributed throughout the small intestine and in surprisingly high numbers in the colonic and even the rectal mucosa [14]. The newly released GLP-1 peptide occurs, depending on species, in an amidated (7–36 amide) or glycine-extended (GLP-1 (7-37)) form. Both forms are rapidly degraded by the serine protease dipeptidyl peptidase-4 (DPP-4), resulting in the generation of the truncated peptides GLP-1 (9–36 amide) and GLP-1 (9–37) [15]. In addition, the neutral endopeptidase 24.11 (neprilysin) also cleaves bioactive GLP-1 [16,17]. GLP-1 exerts its physiological effects by binding to the GLP-1 receptor; this requires intact N-terminal moieties, rendering GLP-1 (9–36 amide) and GLP-1 (9–37 glycine extended) biologically inactive.

## 3. Physiological Effects of GLP-1

The GLP-1 receptor (GLP-1r) belongs to the class 2 family of G-protein-coupled receptors and typically couples to adenylate cyclase via the stimulatory G protein Gs [18]. The receptor is widely expressed in several tissues, including the pancreas, gastrointestinal tract, heart, lungs, kidneys and the peripheral and central nervous system [19,20]. This wide distribution of the receptor contributes to the diversity of GLP-1’s physiological effects. GLP-1 plays a key role in maintaining glucose homeostasis through potentiating glucose-stimulated insulin secretion, expanding pancreatic β-cell mass by increasing proliferation and differentiation [21,22], alongside inhibiting glucagon secretion [23]. It also delays gastric emptying and inhibits gastric acid secretion actions on the CNS [24,25,26,27,28]. Another well-described CNS action is increased satiety and GLP-1 can facilitate weight loss [29,30]. This gut–brain axis maintains the ileocolonic feedback loop, the so-called “ileal brake”, which ensures that the rates of transit and delivery of nutrients are optimal for digestion and absorption. Additionally, GLP-1 may aid intestinal absorption by increasing the mucosa area; indeed, multiple animal studies have reported increased intestinal weights following GLP-1 analogue treatment in mice [31] and rats [32] and have shown the trophic mechanism to be divergent from the action of GLP-2 [33]. Given this, it has also been reported that GLP-1 can potentiate the well-known intestinotrophic effect of GLP-2, suggesting a signal interaction [33]. GLP-1 mimetics have been shown to affect gut permeability in rats [34] and induce barrier-protective effects by enhancing Brunner’s gland function [35]. Moreover, treatment with the GLP-1 mimetic exendin-4 has been shown to moderate the enteric immune response by reducing the production of pro-inflammatory cytokines [36], chiefly due to GLP-1’s ability to downregulate NF-κB phosphorylation and nuclear translocation, effectively reducing the production of proinflammatory cytokines [37]. Furthermore, GLP-1 has been found to have systemic anti-inflammatory effects on the liver, vascular system, heart, brain, kidney, lung, testis and skin [38].

## 4. GLP-1 and Inflammatory Bowel Disease

IBDs, including Crohn’s disease (CD) and ulcerative colitis (UC), are a group of multifactorial disorders characterized by repeated cycles of chronic inflammation and immune cell infiltration, ultimately leading to the destruction of the affected parts of the gastrointestinal tract [39]. CD can affect any part of the intestine, from the oral cavity to the rectum, and manifests in multiple layers of the tissue adjacent to healthy tissue [40]. Contrastingly, UC affects only the colon, usually beginning at the rectum and spreading proximally in the mucosa [41]. Currently, the etiology of IBD remains elusive but is linked to a western lifestyle [42]. Recently, the incidence of IBD has increased in developing countries, threatening to become an emergent global disease [42]. A key treatment goal of IBD is the promotion of mucosal healing, which is a prognostic parameter of clinical remission [43]. Classically, IBD therapeutics fall into three classes: anti-inflammatory, immunosuppressive or biological agents [44]. However, severe IBD may only be treated by surgical removal of the damaged intestine. These surgical resections can then lead to further complications, as in the case of SBS. Given GLP-1’s anti-inflammatory effects, it has been investigated in several pre-clinical studies involving models of IBD.

In 2013, as part of a study investigating the levels of GLP-2 in inflamed colonic tissue, GLP-1 was measured in a mouse model of IBD [45]. IBD was induced by the injection of BALB/C CD4+ T cells into mice with severe combined immunodeficiency (SCID), and resultantly, the tissue level of GLP-1 in the colon was decreased approximately six and nine-fold compared to the SCID and BALB/C controls, likely due to destroyed or suppressed L-cells in the inflammatory state. The authors hypothesized that replacement of the simultaneously lost GLP-2 could be a therapeutic agent for IBD, but it was not until GLP-1’s immunomodulatory actions were established that GLP-1 was considered a therapeutic agent for IBD independently of GLP-2 [46]. Subsequently, Yusta et al. 2015 [36] aimed to evaluate the importance of GLP-1 signaling in a localized inflammatory setting with the use of the GLP-1r knockout (GLP-1r KO) mouse and a dextran sodium sulfate (DSS)-induced colitis model of UC. Female GLP-1r KO mice treated with 3% DSS for seven days had increased sensitivity to intestinal injury, exemplified by increased weight loss, increased disease activity index and greater colonic epithelial damage compared to their wildtype littermates. Moreover, GLP-1r KO mice + DSS had dysregulated gene expression in trefoil factor-3 and interferon-gamma but increased transforming growth factor beta-2 expression. Collectively, these results suggest that endogenous GLP-1 ameliorates the course of DSS-induced colitis, most likely by altering genes involved in immune regulation, epithelial protection and repair; these actions would be preferable to the immunosuppressant mode of action characteristic of current therapies to treat IBD, which can expose the patients to adverse effects such as infection. In the same study, the acute effects of GLP-1 treatment of DSS-induced colitis were investigated in male C57BL/6 mice receiving two subcutaneous injections of the GLP-1 agonist exendin-4 (10 nmol/kg) 12 h apart following four days of DSS. Despite the former observations, exendin-4 treatment failed to affect body weight, colon length or colonic damage score but did reduce colon weight (Table 1). Although more stable than GLP-1, exendin-4 is still rapidly eliminated in mice; the exposure to the peptides may have been insufficient. Otherwise, the results could suggest a sex-mediated difference in GLP-1’s mode of action during DSS-induced colitis in mice; indeed, male mice have been shown to develop a more aggressive form of the disease [47]. However, the timing of exendin-4 interventions could have contributed, since already on day 4, mice began to show signs of increased disease activity. The authors attempted to administer exendin-4 from the beginning of the DSS treatment but found that the results were confounded by agonist-mediated reductions in water, and therefore DSS intake, which consequently is a major limitation of assessing GLP-1’s role in this model of IBD. Contrastingly, a later study conducted by Bang-Berthelsen et al. [35] utilizing a different IBD mode, injection of BALB/C CD4+ T cells into SCID mice, showed that treatment with the long-acting GLP-1 agonist liraglutide (0.6 mg/kg) ameliorated colitis by significantly improving colon weight to length ratios, lowering the histopathological score and reducing pro-inflammatory cytokine levels (Table 1). Interestingly, these improved outcomes were not present following 1mg/kg liraglutide treatment, likely due to the increased body weight loss seen in these animals, which approached critical levels and may have affected the overall health of the mice, counteracting the positive effects seen for 0.6 mg/kg liraglutide. Given GLP-1’s ability to reduce body weight [48], as assessed in obese patients with type 2 diabetes, further studies will be required to approximate the GLP-1 dose needed to improve intestinal outcomes without being offset by weight loss.

The positive results were recapitulated in another rodent study, whereby treatment with sterically stabilized phospholipid micelles coated with GLP-1 (GLP-1-SSM) (15 nmol/100 μL) for one week ameliorated 3% DSS-induced colitis in C57BL/6J mice, by partially attenuating body weight loss, improving stool consistency and alleviating histological changes [49]. Moreover, GLP-1-SSM treatment reduced the expression of the pro-inflammatory cytokine IL-1β and inhibited the decline in the chloride anion exchanger DRA, which plays an important role in mitigating IBD-associated diarrhea (Table 1). A further study showed that during induced colitis (2.5% DSS), oral gavage of lipopolysaccharide (LPS) (5 mg/animal) increased plasma GLP-1 [50]. The effect was dependent on activation of toll-like receptor 4 and the authors suggested that the intestinal injury facilitated access of LPS to the basolateral location of the L-cells, reaching toll-like receptors, resulting in hormone secretion. Toll-like receptor 4 recognizes pathogen-associated molecular patterns, and receptor activation leads to the synthesis of pro-inflammatory cytokines and chemokines characteristic of the innate immune response [51]. These results suggest that L-cells may act not only as nutrient sensors but also as sensors of intestinal injury, responding with GLP-1 secretion, which may be an early biomarker of gut injury; however, these findings need further consolidation in human gastrointestinal damage. In addition, it is notoriously difficult to measure GLP-1 in mice, rendering the involvement of the L-cells uncertain [52].

Currently, there is one published case report exploring GLP-1 treatment in a patient with ulcerative colitis [53]. In this case, administration of daily subcutaneous liraglutide injections (0.6 mg titrated to 3.0 mg) resulted in full remission of colitis symptoms with minimal adverse effects, limited to mild nausea associated with dose titration (Table 1). Despite the lack of studies of GLP-1 treatment in human IBD conditions, promising evidence from animal experiments warrants further investigation into the human conditions.

## 5. GLP-1 and Short Bowel Syndrome

In adults, SBS can anatomically be defined as possession of less than 200 cm of functional small intestinal length [54]. In children, it can be defined as a need for intravenous nutritional supplementation or, in neonates, a remaining intestinal length less than 25% of that corresponding to gestational age [54]. SBS is a malabsorptive condition most frequently seen in patients with one or multiple surgical resections, leading to chronic intestinal failure (CIF) [55]. CIF arises due to the reduced absorptive capacity of the remnant bowel and can be exacerbated, particularly in SBS patients with distal bowel resections, by the disruption of the ileal brake mechanism, which leads to rapid gastrointestinal motility after food intake, gastrointestinal hypersecretions and a further decreased absorptive capacity [56,57]. SBS and/or intestinal failure can lead to a reliance on parenteral support [55], which, in the long-term, is associated with liver complications and catheter-related infections [5].

Amongst other clinical and anatomical features, SBS can be classified into two subcategories: patients with an intact colon in continuity and those lacking [54]. In general, the preservation of the colon is a beneficial factor that contributes to the adaptive process following resection. Indeed, in a small case report of 17 patients with a remnant bowel of 100 cm or less, 12 patients required parental support, whilst of the 21 patients with a remnant bowel of 50 cm and a colon in continuity, only seven patients required parental support [58]. Such observations drove the hypothesis that the adaptive process could be positively influenced by the increased plasma concentration of GLP-2, which was a known intestinotrophic hormone [59], released from the L-cells in the colon [60]. In 2000, a small case–control study conducted by Jeppesen et al. showed SBS patients with a preserved colon (all < 140 cm, four with jejunoileo-colic anastomosis but with <10 cm ileum and three with jejuno-colic anastomosis) to have a three-fold fasting and 1.8-fold post-prandial increase in plasma GLP-2 compared to healthy, age-matched controls [60]. The group also measured GLP-1 and described a similar two-fold fasting and 1.3-fold post-prandial increase in plasma GLP-1 compared to controls.

Eleven years later, following the then recent approval of the GLP-1 receptor agonist exendin-4 for the treatment of type 2 diabetes [61], exendin-4 was given in a small retrospective study in SBS patients [62]. The study aimed to assess the influence of exendin-4 on gastric emptying since patients lacking the whole or part of the ileum have unregulated gastric and proximal small intestinal transit. In the study five SBS patients all with ≤ 90 cm remnant small bowel, four with colon in continuity, received exendin-4 twice daily by subcutaneous injection, at a dose of 5 µg, for one month. Antroduodenal manometry was performed in two of the patients and all patients were asked to complete a stool diary for the remainder of the study. In both cases, manometry findings showed that exendin-4 decreased antral contractions in either the fasting or fed state, therefore slowing gastric emptying and facilitating a greater contact time for chyme with the absorptive mucosa surface (Table 1). Resultantly, all five patients showed improvements in bowel frequency and form, and three of the patients no longer required total parenteral nutrition. In this small group, exendin-4 had minor adverse effects, with only one patient reporting nausea, which was resolved following dose reduction. Correspondingly, in another open-label, placebo-controlled trial, nine SBS patients (164–64 cm) with end-jejunostomies (2 with colon continuity) were admitted four times and either received a 72-h infusion of synthetic GLP-1 (7-36), placebo (saline), GLP-2 and GLP-2 + GLP-1 (1 pmol/kg/min) followed by a 30-day washout period [63]. GLP-1 infusion reduced diarrhea and fecal excretion, albeit to a lesser extent than GLP-2; however, the combined infusion of GLP-1 and GLP-2 had additive effects on intestinal absorption compared to either peptide alone (Table 1). Interestingly, the combined infusion of GLP-1 and GLP-2 also appeared to lower the experience of adverse effects of nausea and decreased appetite. Together, these results highlight the potential of a dual agonist not only to improve absorption more effectively than monotherapy, thereby reducing the initial required dose but also to mitigate the dose-dependent adverse effects of GLP-1. Currently, Zealand Pharma is developing a long-acting GLP-1R/GLP-2R dual agonist, ZP7570, designed for the treatment of SBS and aimed at exploiting the intestinotrophic benefits of GLP-2 and the gastrointestinal motility benefits of GLP-1 [64]. ZP7570 is currently part of a randomized, double-blind, placebo-controlled, phase 1 clinical study in healthy subjects that was initiated in June 2019, and animal data from preclinical experiments with the compound are yet to be published [64].

Pre-clinical studies utilizing GLP-2 showed significant effects on the intestinal mucosa, which was of great interest to the pharmaceutical industry [59]. Indeed, following the USA Food and Drug Administration (FDA) and European Medicines Agency (EMA) approval, the GLP-2 analog teduglutide has been commercially available since 2012 in adults and since 2019 in pediatric patients 1 year of age and older, for the treatment of SBS in patients requiring parenteral nutrition [65]. The current annual cost of teduglutide is USD 300,000 per patient [66]. In context, treatment of SBS patients in Denmark with teduglutide would equate to 6% of the national medicine budget for a syndrome that affects an estimated 0.01% of the population [67]. Given this, other price-competitive treatments to teduglutide are being explored, such as liraglutide. In this respect, the GLP-1 analogue liraglutide, which was approved for the treatment of type 2 diabetes in Europe in 2009 and the USA in 2010, is 250 times cheaper than teduglutide, at an annual cost of USD 1200 per patient [67]. Additionally, liraglutide may compensate for the lost ileal-break mechanism, improving the accelerated gastrointestinal motility [56] and gastrointestinal hypersections seen in SBS patients [57]. In a single-center, open-label, non-controlled pilot study, eight patients with end-jejunostomy and dependence on parenteral support attended two 72-h metabolic balance studies, one before and one after eight weeks of subcutaneous liraglutide treatment (dose week 1–2, 0.6 mg/day and week 2–8, 1.8 mg/day) [67]. Comparative to baseline, liraglutide treatment reduced ostomy wet weight output by approximately 16% and improved wet weight absorption approximately 12-fold, resultantly increasing urine output by 40%, which was interpreted as improved hydration status (Table 1). The effects of liraglutide on electrolytes and creatinine were minimal, but intestinal energy absorption increased by 9%, with increases most prominent in carbohydrate and protein, compared to lipid. Interestingly, gastric emptying measured by plasma paracetamol was not affected by liraglutide. This contrasted the experiences of the patients, who reported increased delays from oral intake to onset of ostomy output. Additionally, enterocyte mass was not affected by liraglutide treatment, in contrast to what has previously been shown in experimental animals [31]. Given these discrepancies and the limited study size, larger controlled trials are warranted to consolidate the efficacy of liraglutide; yet, given the high cost of phase 2 and 3 clinical trials and the small orphan population of SBS patients, realization of these trials will prove challenging.

## 6. GLP-1 and Intestinal Toxicities

Intestinal toxicity is a common complication of anti-neoplastic therapies such as chemo- and radiotherapy and the toxic reactions are most often referred to as mucositis. The pathophysiology is multifactorial but the accepted explanation is a process starting with the production of reactive oxygen species due to DNA damage, resulting in upregulation of transcription factors such as NFkB and activation of pro-inflammatory cytokines and matrix metalloproteinase [68]. This leads to cell death and tissue destruction. Eventually the mucosa will recover. Mucositis can occur anywhere in the gastrointestinal tract, but only intestinal mucositis has been investigated in relation to GLP-1. The patients present with abdominal pain and diarrhea and have an increased risk of infection due to impaired intestinal barrier function and neutropenia because of the cytotoxic therapy. Severe mucositis can have both clinical and economic implications, including the need for total parenteral nutrition, hospitalization and reduction in anti-cancer therapy [69]. Therefore, the development of intervention strategies that can reduce the complications of anti-cancer agents is vital, and recently, GLP-1 has attracted attention as a potential solution.

During mucositis, GLP-1 has been suggested to restore the intestinal absorptive capacity through the restoration of the mucosa area. Indeed, multiple animal studies have described a tropic effect in the intestine following GLP-1 agonist treatment [32,33] and GLP-1 was increased following the induction of mucositis in mice [70] and after chemotherapy in humans [71]. One study, using the administration of 5-fluorouracil (5-FU) in a mouse as a model of chemotherapy-induced mucositis, showed GLP-1 to ameliorate mucositis-associated pathology [70]. Subcutaneous injection of liraglutide (600 µg day—2 to 1, 300 µg day 1 to 2) twice daily abolished the chemotherapy-associated decrease in intestinal weight, prevented the reduction in the mucosal area in the duodenum and jejunum and decreased disease activity in the treated mice [70]. Moreover, in the same study, subcutaneous injection with 25 µg of the GLP-1 antagonist exendin (9–39) twice daily delayed small intestinal weight recovery after chemotherapy [70] (Table 1). These results not only suggested a beneficial role of GLP-1 following acute injury but also suggested that GLP-1 may also play a role in recovery. In contrast to these results, in another study from 2018 by Hytting-Andreasen et al. on a low dose of GLP-1 receptor agonist in treatment in the same model of mucositis [72], exendin-4 (12.5 µg subcutaneous twice daily) failed to protect against the chemotherapy-associated loss in SI weight and villus atrophy at day three (Table 1). Additionally, the same protocol repeated in L-cell deficient mice showed that exendin-4 also failed to protect against the chemotherapy-associated body weight and SI weight loss at five days. These conflicting results show a mixed effect of GLP-1 treatment in chemotherapy-induced mucositis, perhaps due to the low dose or insufficient exposure, which warrants further clarification. Interestingly, these studies also investigated co-treatment of GLP-1 and GLP-2 in chemotherapy-induced mucositis compared to the GLP-1 and GLP-2 monotherapy. Exendin-4 (12.5 µg twice daily) and teduglutide (12.5 µg twice daily) treatment of the mice, sacrificed on day three, prevented villus atrophy and reduced body weight and SI weight losses, and the combined treatment was superior to GLP-2 monotherapy [72]. Furthermore, when studied at day five, only co-treatment prevented severe body weight loss after chemotherapy, and co-treatment protected against SI weight and mucosa mass loss through hyperproliferation, again superior to GLP-2 monotherapy. This suggests a signal interaction between the glucagon-like peptides that can benefit both the acute and recovery phases of chemotherapy-induced mucositis. Furthermore, GLP-1 (in the presence of GLP-2) may also influence systemic parameters, evidenced by its necessity to prevent severe body weight loss in the mice after chemotherapy. Development of dual GLP-1 and GLP-2 agonists has already begun and treatment with the novel co-agonist GUB09-123 has shown superiority to teduglutide with respect to increasing gut volume and mucosal area in mice [73]. Presently, the molecular synergistic mode of action of the two peptides is unknown [70], yet they are thought to act independently; at least, neither liraglutide nor exenatide stimulates cAMP release from GLP-2 receptor transfected cells [31]. Given that GLP-1 has been shown to potentiate the signaling of the growth factor IGF-1 [74], it could be speculated that this could be a mechanism to amplify the signal upon dual exendin-4 and teduglutide treatment, but this is yet to be established.

Following the promising results of GLP-1 and GLP-2 administration after chemotherapy, it was hypothesized that GLP-1 could be an early marker of gut injury in patients receiving chemotherapy [71]. An uncontrolled trial of 66 patients undergoing high-dose chemotherapy and autologous stem cell transplantation showed increased fasting levels of plasma GLP-1 and, importantly, that high GLP-1 levels shortly after high-dose chemotherapy were predictive of systemic inflammation markers during the first three weeks after transplantation. The results suggest that GLP-1 plays an important role in mucosal defense. Indeed, GLP-1 has been found to interact with intestinal intraepithelial lymphocytes that express GLP-1 receptors, reduce the production of pro-inflammatory cytokines [36] and improve Brunner’s gland function, all of which may contribute to improving mucosal integrity [35]. Therefore, it may be speculated that the increased plasma GLP-1 following chemotherapy may help to limit inflammation and tissue damage.

## 7. GLP-1 and Coeliac Disease

Coeliac disease (CD) is an autoimmune disorder triggered by the ingestion of gluten in genetically predisposed individuals [75]. The mainstay of CD treatment involves adhering to a gluten-free diet; however, multiple economic, social or societal factors can affect diet [75]. This has fueled research into alternative non-diet therapies. Primarily, it affects the small intestine, with classic symptoms including diarrhea, abdominal distention, malabsorption and loss of appetite, and the condition can lead to growth abnormalities in children [76]. Typical histological changes include villous atrophy, crypt hyperplasia and chronic inflammation of the lamina propria [77]. Given these alterations in the mucosa, GLP-2, and subsequently GLP-1, were suggested to have beneficial effects on CD pathology.

Several human studies have described altered plasma GLP-1 profiles in patients with CD with contrasting outcomes. In 2006, Caddy and colleagues conducted a small controlled trial (12 CD patients, 7 age-matched healthy controls) and found no differences between basal and post-prandial (gluten-free) plasma GLP-1, between patients with confirmed CD and healthy controls [78]. In contrast, in 2014, Papastamataki et al. showed GLP-1 to be lowered in a pre-prandial state in CD as part of a controlled trial in children aged 7–12 (34 CD patients, 18 sex- age- and body mass index-matched healthy controls) [79]. Discrepancies between the studies may be due to differences in study size, weight matching of controls, the assay used to measure GLP-1 and the age of the participants. However, a cohort study conducted in children aged 5–18 showed also no differences between basal or peak (following oral glucose tolerance test) plasma GLP-1 levels in patients with CD, when compared to patients without CD histology but still referred for esophago-gastro-duodenoscopy due to gastrointestinal tract symptoms [80]. The authors also investigated GLP-1r expression in the duodenal tissue and found no differences between CD and non-CD patients. Taken together, these results demonstrate the complex landscape between CD and GLP-1, which combined with the lack of established animal models for CD, means that the role of GLP-1 in coeliac disease is currently unknown.

## 8. Theoretical Drug Safety of GLP-1

Given the demonstrated intestinotrophic properties of GLP-1 [31,32], it has been essential to assess if exogenous GLP-1 administration is linked to the progression of subclinical malignancies. Indeed, it has been shown that exendin-4 treatment enhanced β-cell proliferation in the pancreas by influencing the wnt/β-catenin pathway, which is also associated with colon tumorigenesis [81]. However, in a mouse model of colorectal cancer, induced by 1,2 dimethylhydrazine injections for 12 weeks, mice treated with liraglutide (300 μg, twice daily for 45 days) showed no increase in the number of aberrant crypt foci, mucin-depleted foci or total adenomas [31] (Table 1). Similarly, another study utilizing exendin-4 (10 nmol/kg,) and a genetic mouse model of colon cancer (ApcMin/+ mice) found no effect on the number and size of aberrant crypt foci or adenoma load in the colon [33] (Table 1). The same study assessed GLP-1r expression from human colorectal tumors and found the mRNA transcripts to be downregulated in the colorectal tumors compared to adjacent non-neoplastic tissue [33]. Furthermore, in a large interventional study of 9341 participants with type 2 diabetes, liraglutide treatment did not increase neoplasm incidence [82]. This conclusion was supported by a meta-analysis including 50,453 patients from randomized controlled trials, assessing the incidence of neoplasms in type 2 diabetic patients receiving GLP-1 receptor agonists compared with placebo or other hypoglycemic drugs; in this study, there was no increase in malignant neoplasm formation upon GLP-1 agonist use [83].

Following the approval of exendin-4 for the treatment of patients with type 2 diabetes, the FDA began to receive reports suggesting a link between acute pancreatitis and exendin-4 treatment, which was to be supported by animal data [84]. Resultantly, both the FDA and the EMA undertook evaluations of the post-marketing reports of pancreatitis and pancreatic cancer in patients using GLP-1-based drugs (41,000 participants) and re-evaluated existing and performed new toxicology reports to investigate pancreatic adverse events [85]. Both agencies preliminarily concluded that there was no link between incretin-based drugs and pancreatitis or pancreatic cancer, but pancreatitis would continue to be considered a risk associated with these drugs until more data are available.

When evaluating GLP-1’s therapeutic potential with respect to intestinal disease, decreased body weight can be considered as an adverse effect. High-dose liraglutide (3.0 mg) was found to reduce body weight by a mean of 8.4 ± 7.3 kg in patients with a BMI of at least 30, or least 27 if they had treated or untreated dyslipidemia or hypertension, compared to placebo, as part of a large, randomized, controlled study involving 3731 participants [29]. Furthermore, similar benefits were seen in obese adolescents [30]. Subsequently, liraglutide has been FDA-approved for chronic weight management of obese adults and children above the age of 12 [86]. The approved dose for weight loss is a high dose of 3.0 mg and the body weight clearly decreases in a dose-dependent manner [87]. Given this, any consideration of the treatment of intestinal diseases with GLP-1 agonists would have to carefully consider the therapeutic dose to avoid undesired weight loss.

High-dose GLP-1r agonist treatment can also lead to transient nausea, the most frequently described adverse effect of GLP-1r agonists (~30–50% of patients) [88]. Despite nausea being a relatively mild adverse effect, it can lead to medication discontinuation. The prevalence of this nausea varies between the agonists prescribed, with reductions seen in patients switched from the short-acting exendin-4, which has a peak concentration 2–3 h after injection, to an agonist with a flatter pharmacokinetic profile, such as medium-acting liraglutide [89]. Furthermore, the experience of nausea decreases when doses are slowly titrated up to the therapeutic dose [90] and is usually only associated with the early days of new dose treatment. Despite this, nausea would be an unwanted adverse effect in the treatment of patients with intestinal disease, who might already have a reduced capacity for oral or enteral nutrition. Given this, doses would have to be slowly titrated and lower doses of agonists with flatter pharmacodynamic profiles should preferentially be used.

A less common (~10–20% of patients) but potentially severe adverse effect in patients with intestinal disease is the presence of diarrhea [91]. In contrast to nausea, diarrhea was experienced more frequently in patients receiving long-acting agonists compared to short-acting [8]. Additionally, prolonged treatment, in either case, did not diminish the effect [92]. Constipation was also recorded in ~7% of participants treated with liraglutide, ~11% of patients treated with long-acting exendin-4 and ~ 6% of patients treated with exendin-4 [91,93]. Given these relatively common adverse effects in the GI tract, GLP-1 would have to be prescribed with extreme caution to patients with intestinal diseases, so that medication does not aggravate the clinical symptoms presented. Additionally, the adverse effects in the GI tract could limit the compounds from being used in the most efficacious manner, which could reduce therapeutic potential.

## 9. Conclusions

The need for treatments of intestinal diseases to go beyond symptomatic management is increasing, as the field strives to further improve patients’ quality of life, reduce the high economic impact on healthcare systems and mitigate the societal impact of these diseases. This, combined with the increase in the global prevalence of intestinal diseases [94], has inspired research to explore novel treatments that promote intestinal rehabilitation. Despite “intestinal disease” covering a broad range of gastrointestinal disturbances, those covered in this review (IBD, SBS, intestinal toxicities and coeliac disease) share some common features of mucosa atrophy, decreased mucosal integrity, increased inflammation as well as having GLP-1 explored as a therapeutic to ameliorate the disease-associated pathology. Promising outcomes from animal data have led to exploration in small human trials in the case of SBS and IBD with encouraging results, but larger studies will be necessary to corroborate and consolidate these outcomes. Studies of intestinal toxicities such as chemotherapy-induced mucositis have highlighted the potential of dual GLP-1 and GLP-2 agonists to improve not only local intestinal but also systemic parameters in animals following chemotherapy. However, GLP-1’s action alone is still to be clarified, but human studies have suggested GLP-1 to be predictive of mucosa rehabilitation. The lack of established animal models recapitulating coeliac disease, combined with conflicting human data, means that the role of GLP-1, if any, is yet to be established.

Irrespective of the potential benefits of GLP-1-based therapy on intestinal diseases, the adverse effects of GLP-1r agonists should not be ignored. Despite the compounds being safe and tolerable for patients with type 2 diabetes, the gastrointestinal adverse effects are of more concern to patients with intestinal diseases. Should GLP-1 based therapies be attempted, they should be slowly titrated, remain at low dosing, and the variant (short-acting vs. long-acting) should be chosen to suit the individual needs of the patient. This will provide challenges in balancing the adverse effects with the compounds’ efficacy. These problems may possibly be eased by dual GLP-1 and GLP-2 agonists currently in development or by using GLP-1 agonists as part of multimodal treatment strategies, but this is yet to be elucidated.

## Figures and Tables

**Table 1:** Summary of the reviewed studies where GLP-1 or GLP-1 mimetics are used as treatment for intestinal disease.

Drug	Dose	Model/Patient Group	Therapeutic Effects	Suggested Mechanism	Ref.
**Exendin-4** 39-aa peptide; DPP4 resistant	10 nmol/kg SC two injections 12 h apart	C57BL/6 mice with DSS induced colitis	Reduced colon weight	Moderation of the enteric immune response by reducing the production of pro-inflammatory cytokines	[36]
	5 µg × 2 SC for 1 month	SBS patients with ≤90 cm remnant (*n* = 5)	Improved bowel frequency and form and three of the patients no longer required total parenteral nutrition	Liraglutide decreased antral contractions and therefore facilitated a greater contact time for chyme with the absorptive mucosa surface	[62]
	12.5 µg × 2, SC (alone or in combination with GLP-2)	C57BL/6J mice with 5-FU induced mucositis	Exendin-4 alone failed to protect against injury. Combined treatment prevented villus atrophy and BW and SI weight losses. Combined treatment was superior to GLP-2 monotherapy		[72]
	10 nmol/kg	ApcMin/+ mice	No effect on the number and size of aberrant crypt foci or adenoma load in the colon		[33]
**Liraglutide** Acylated GLP-1 enabling albumin binding	0.6 mg/kg × 1 SC for 35 days	SCID mice with BALB/C CD4+ T cells induced colitis	Improved colon weight to length ratios; lowered the histopathological score and reduced pro-inflammatory cytokine levels	Upregulation of the barrier layer cytokines and chemokines (IL-33, CCL20 and MUC5B)	[35]
	0.6 mg titrated to 3.0 mg ×1 SC for 30 days	IBD patient (*n* = 1)	Full remission of colitis symptoms (case report)		[53]
	Week 1–2 0.6 mg × 1 and week 2–8 1.8 mg × 1, SC for 8 weeks	SBS patients with end-jejunostomy (*n* = 8)	Reduced ostomy wet weight output and improved wet weight absorption and hydration status		[67]
	3.6 mg × 2 (3 days) 1.8 mg × 2 (2 days) SC for 5 days	C57BL/6J mice with 5-FU induced mucositis	Abolished the loss of SI weight, prevented the reduction in the mucosal area in the SI and decreased disease activity	Proliferative effects possible involving IGF-1R	[70]
	300 μg × 1, SC for 45 days	C57BL/6J mice with DMH induced colonic neoplasia	Treatment showed no increase in the number of aberrant crypt foci, mucin-depleted foci or total adenomas		[31]
**GLP-1-SSM** Sterically stabilized phospholipid micelles coated with GLP-1	15 nmol × 1 IP for 7 days	C57BL/6J mice with DSS induced colitis	Attenuated BW loss, improved stool consistency and alleviated histological changes	Increase in expression of pro inflammatory cytokine IL-1β was partly reversed by GLP-1-SSM, indicating anti-inflammatory effects	[49]
**GLP-1 (7-36)** 29 aa peptide	1 pmol/kg/min IV for 72 h (alone or in combination with GLP-2 (1–33))	SBS patients (164–64cm) with end-jejunostomies (2 with colon continuity) (*n* = 9)	GLP-1 infusion reduced diarrhea and fecal excretion, albeit to a lesser extent than GLP-2; however, the combined infusion of GLP-1 and GLP-2 had additive effects	Restoration or reduction in secretions and motility of the proximal bowel	[63]

Abbreviations: DSS = dextran sulphate sodium, BW = body weight, IBD = inflammatory bowel disease, SBS = short bowel syndrome, 5-FU = 5-fluorouracil, SI = small intestine, DMH = dimethylhydrazine.
